# The Effect of Blindness on the Development of Fear Response in Chickens of Different Ages

**DOI:** 10.3390/ani16203180

**Published:** 2026-10-10

**Authors:** Kimberly M. Cheng

**Affiliations:** Avian Research Centre, Applied Animal Biology Program, Faculty of Land and Food Systems, University of British Columbia, Vancouver, BC V6T 1Z4, Canada; kimberly.cheng@ubc.ca

**Keywords:** blindness, fear response, tonic immobility, sex, age, prior experience, testing time, chicken

## Abstract

The objective of this study was to examine the effect of blindness on the ontogeny of fear response. We used a genetically blind chicken model (*rc* chickens) and tested fear response based on tonic immobility (TI). There were four blind groups and four sighted groups of *rc* chickens in this study, and each group consisted of 45 chickens. We performed the TI test on days 2, 14, 28, and 70 to study the ontogeny of fear response. Blind chickens were more difficult to induce into a TI response and stayed in TI for a significantly shorter time compared to sighted controls. The results from the TI tests indicated that the blind males had a slower rate of developing a TI response (from Day 2 to Day 70) than blind females. Blindness in *rc* chickens is caused by a deletion in the *GC1* locus, resulting in the disruption of cGMP metabolism. Besides causing the degeneration of rods and cones in the retina, cGMP deficiency may have a direct effect on brain cells, thereby suppressing tonic immobility. cGMP also seems to affect male sexual maturity differently than female sexual maturity. Our results indicated that the ontogeny of fear response was dependent on the interaction of age, sex, prior experience and visual input, and the ontogeny of TI response in *rc* chickens may not solely reflect the ontogeny of the fear response but also the physiological processes affected by the gene mutation.

## Simple Summary

The objective of this study was to examine the effect of blindness on the development of fear response in chickens. We used a genetically blind chicken model (*rc* chickens) and tested fear response based on tonic immobility (TI). TI tests were administered to male and female blind and sighted *rc* chickens on day 2, day 14, day 28, and day 70 after hatching. Blind birds were more difficult to induce into TI and stayed in TI for a significantly shorter time compared to sighted controls. The results from the TI tests indicated that the blind males had delayed maturity and a slower rate of developing a TI response than blind females and sighted birds. Because the *rc* mutation also affects physiologic processes other than blindness, differences in TI between blind and sighted *rc* chickens cannot necessarily be attributed solely to the absence of visual input.

## 1. Introduction

Domestic chickens are primarily housed in high-density conditions. The stress of such a situation can result in poor animal health and decreased production. Earlier researchers interested in animal welfare have studied fearfulness in chickens under different housing conditions. Indications were that levels of auditory stimulation [1] and visual stimulation [2,3] affect levels of fearfulness.

Tonic mobility (TI) is a commonly used test for fearfulness in many animals [2,4,5,6]. A long duration of TI response is associated with high levels of fear in potentially frightening situations [7,8]. In laboratory conditions, the duration of the TI response is defined as the latency of righting time, and it is considered an indicator in the assessment of the fear response [9,10]. TI response has been studied extensively. Several factors have been identified as TI-modifying factors that affect the duration of the TI response: method of induction [11,12], visual stimuli [9,13,14,15,16], age [17,18,19], sex [1,9,20], previous experience [9,12], social experience [19,21,22], housing structure [2,3,21], genetic background [23,24], nutrition [25,26], and noise [9,27].

Vision plays a major role in gathering information for birds; “birds are the most visually dependent vertebra” [28]. Visual stimuli plays an important role in the elicitation of tonic immobility response. The work of several researchers has focused on the physical proximity of potential predators: the eyes of the predators or eye-like shapes [29] (https://blog.londolozi.com/2025/06/16/a-glimpse-into-the-wild-predator-and-prey-through-different-eyes/, accessed on 11 September 2026). Campo et al. [27] determined that restricted vision does result in lowered levels of fearfulness. However, the effect of blindness—the total lack of visual stimuli—on chickens of different ages has not been examined.

The *rc* mutation in chickens is a naturally occurring mutation [30]. The retina degenerates during development so that at hatch the chicks are blind and never experience normal vision. The existence of these genetically blind chickens makes it possible for comparison with normal chickens. The *rc* chickens provide a model to separate the effects of visual and other stimuli on fear.

The objective of this study was to evaluate the effects of Blindness, Age, Sex, Prior experience, and the Time of testing on the development of fear response in domestic chickens using TI and a well-defined genetically blind chicken [30] as a model. The TI response was measured in terms of the number of inductions required, latency of first head and first leg movements, and righting time of birds during the immobility test. The null hypotheses to be tested are as follows: 1. Blindness has no effect on the TI scores of *rc* chickens; and 2. There were no interactions among blindness, age, sex, and prior experience affecting the TI scores of rc chickens.

## 2. Materials and Methods

### 2.1. Experimental Birds

The line of blind chicken was originally established from an experimental line of Rhode Island Red chickens with a single locus autosomal recessive mutation [30]. The chickens with this mutation have degenerated rods and cones in their retina and fail to respond to visual stimulation [31] at hatch and thereafter. The mutation was named *rc* (rods and cones) according to the poultry gene system [30]. Later molecular studies identified that the mutation was a null deletion in the photoreceptor guanylate cyclase (*GC1*) gene [32].

The blind birds used in this study were homozygous (*rc*/*rc*). Sighted heterozygous (*Rc^+^*/*rc*) birds were used as controls. Both types were from *Rc^+^*/*rc* matings, meaning that the blind and sighted birds used in the experiment were very similar in genotypic background, except for the mutation. All birds were exposed to a 14L:10D photoperiod.

### 2.2. Experimental Design

Upon hatching, blind chicks were individually marked, weighed, and randomly assigned to one of four groups (1B, 2B, 3B, and 4B). Each group consisted of 45 individuals. Sighted chicks were treated similarly and assigned to group 1S, 2S, 3S, or 4S. The eight groups were randomly assigned to different sections of two battery brooders placed side by side in the same room. A total of 360 birds was used in the study. Blind chicks quickly learnt where the feed and water were located by using their explorative behaviour (circular walking). Once the birds were 4 weeks old, they were moved to side-by-side 6 m × 6 m floor pens, with each group occupying a pen. All pens were maintained under identical housing and management conditions. TI was induced and measured on individual birds. Because TI was administered and measured at the individual level and no treatments were applied at the pen level, an individual bird was considered the experimental unit for assessment of TI response.

TI was assessed on days 2, 14, 28, and 70 after hatching. Birds from groups 1B + 1S, 2B + 2S, 3B + 3S, and 4B + 4S were evaluated on days 14, 28, and 70, respectively. Individuals in groups 1B and 1S were also repeatedly tested at different ages. Consequently, except on day 2, birds assessed at each age originated from two pens within each genetic type (blind or sighted), including one pen represented with birds of all ages and one age-specific pen. This sampling scheme was adopted to reduce the potential influence of previous TI experience on subsequent behavioural responses, while also allowing for comparisons between previously tested and previously untested birds.

Individual body weights were recorded at hatch, and again on days 28 and 70 following TI testing. All birds were retained until the end of the experiment, at which time the sex of each individual was determined.

### 2.3. TI Test Protocol

#### 2.3.1. Apparatus

**Figure 1:**
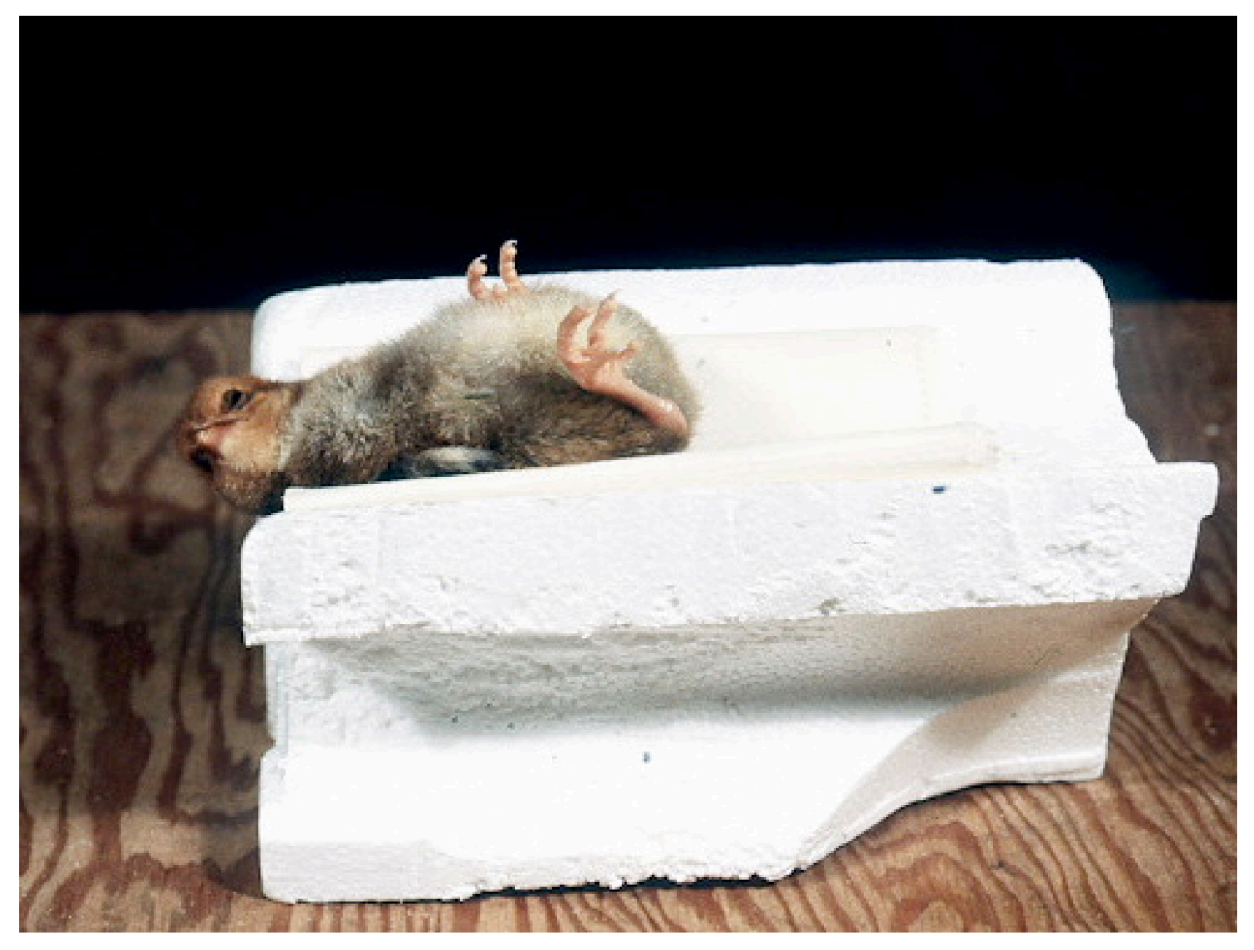
TI test apparatus for Day 2 chicks.

The test apparatus for Day 2 chicks was a Styrofoam platform with a trough in the middle (Figure 1).

**Figure 2:**
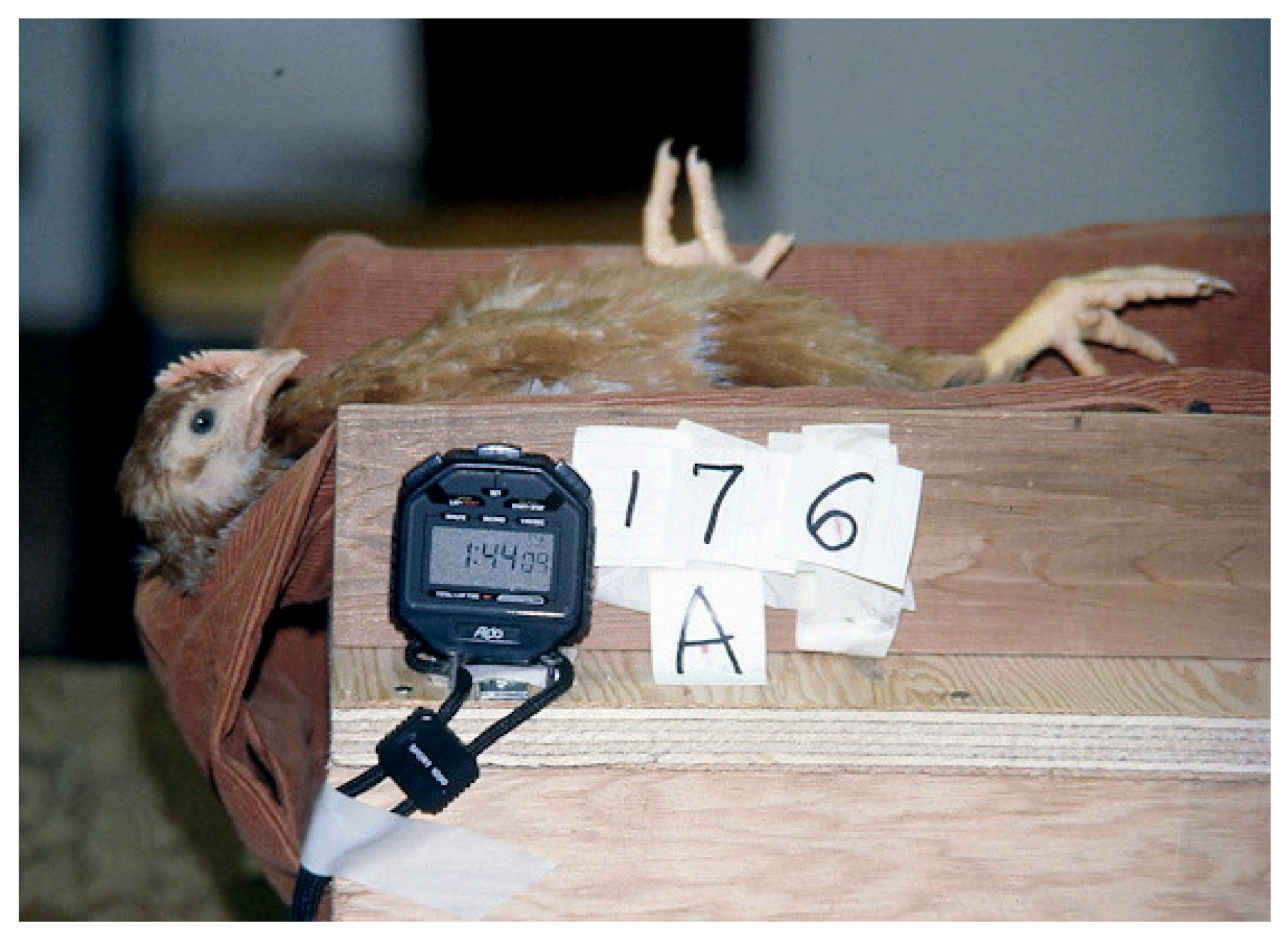
TI test apparatus for Day 14, 28, and 70 chickens.

The test apparatus for older birds consisted of a 30 cm square plywood platform with 4 cm high, width-adjustable V-shaped sides above the platform along one axis (Figure 2). The width of the depression could be adjusted as the birds grew older. The depression was covered by corduroy to cradle the inverted body of the bird. The apparatus was placed on a table at one end of a darkened and visually isolated 6 m × 6 m room adjacent to the rooms where the birds were reared. Auditory contact was not blocked. A 40-watt shaded light was placed about 61 cm above the apparatus so that the apparatus would be lighted but the rest of the room would be dark during testing. A video camera was placed about 3 m away (in the dark) from the apparatus. A digital stopwatch was taped beside the apparatus facing the camera so that time could be recorded. A card with a random number was placed next to the stopwatch, and the number was later keyed to the group number of the bird on a record sheet. During the test sessions, the experimenter was sitting still in the dark corner of the room furthest away from the test apparatus.

#### 2.3.2. TI Test Procedure

The order of testing was randomized. A bird from a pre-determined group was taken from the brooder and hand-carried to the darkened test room. The bird was put on its back on the cradle of the test apparatus, firmly held for 15 s and then released. If the bird stayed on its back for at least another 15 s then the induction was deemed successful. A maximum of 600 s was allowed. If a bird was still under TI at the end of this period, the trial was ended and the bird put back in its brooder. If the bird got up before the end of 15 s (after the release), the induction was considered unsuccessful. The bird would be caught and induced again following the same procedure. A maximum of six inductions was allowed. After six unsuccessful inductions, the bird would be taken back to its home pen and a score of 15 s would be given [33]. All of the testing was conducted by the same researcher, and each trial was video-recorded. Upon reviewing the tapes, the number of inductions required, the latency of first head and first leg movements, and the righting time (i.e., the time required for the bird to right itself with both feet on the platform/table) were recorded. The researcher reviewing the tapes was not the one conducting the tests and was unaware of which group the bird under observation belonged to.

### 2.4. Statistical Analyses

PASW Statistics software (PASW Statistics GradPack 17.0, release 17.0.2., IBM^®^ SPSS^®^, Chicago, IL, USA) was used to analyze the data. The significance level was set as *p* < 0.05.

Experimental results were organized into two data sets, and statistical analyses for each data set were determined based on our research questions, considering selected test assumptions. In order to use parametric tests, three dependent TI response variables (latency of the first head movement, first leg movement, and righting time) were transformed using natural logarithm transformation. Marginal means (in seconds) and SE reported in the Section 3 are the results of the back-transformation from a natural log scale. Transformation did not work on the ‘number of inductions required’ dependent variable, and as a result, non-parametric tests (Kruskal–Wallis and Mann–Whitney tests) were used to analyze the results related to this variable.

#### 2.4.1. Using the First Data Set to Assess the Effects of Age, Blindness, Sex and Time of Testing on TI Responses and Body Weight of Birds

This data set includes the TI responses of individual chicks in groups 1B + 1S, 2B + 2S, 3B + 3S, and 4B + 4S on days 2, 14, 28 and 70 after hatching, respectively (i.e., birds which were tested for the first time at the identified measurement day). Pearson or Spearman’s correlation coefficients were calculated to investigate the relationship between the dependent and independent variables in our study. To assess the effects of Age (in four levels: 2, 14, 28 or 70 days), Blindness (in two levels: blind or sighted) and Sex (in two levels: male or female) on the natural logarithms of three dependent TI response variables (Latency of the first head movement, first leg movement, and righting time), ANOVA tests were applied, and back-transformation of marginal means and SE are reported in the Section 3. Sidak correction post hoc tests were used to follow up any significant main effects or interaction effects with more than two levels.

To assess the differences in the body weight of the birds considering the effects of Age, Sex, and Blindness, a linear model (GLM procedure) was used to conduct a repeated-measures ANOVA separating within-bird effects (weight at three ages: Day 2, 28, and 70) from between-birds effects (i.e., Age, Blindness, and Sex). Sidak correction tests were used as the post hoc test (if required).

To assess the effects of the Time of testing (as an independent variable in three levels: morning, afternoon and evening), ANOVA tests were used on the natural logarithms of three dependent TI response variables (Latency of the first head movement, first leg movement, and righting time). In addition, back-transformation of marginal means and SE is reported in the Section 3.

Kruskal–Wallis and Mann–Whitney tests were also utilized to assess the effects of Age, Blindness, Sex and Time of testing (separately) on the ‘number of inductions required’ variable.

Thus, all age-related comparisons were based exclusively on birds undergoing the TI test for the first time at the respective age, thereby eliminating any potential confounding effects of prior TI test experience.

#### 2.4.2. Using the Second Data Set to Assess the Effects of Experience on TI Responses of Birds (Considering Blindness and Sex Factors)

To investigate the effect of previous TI test experience on the TI response variables, the responses of birds were categorized based on their age (without including second-day data because there were no experienced groups at day 2). Birds in the 1B + 1S groups were included as the experienced birds at all three age categories, while birds in the 2B + 2S, 3B + 3S, and 4B + 4S groups were included as the novel birds at 14, 28, and 70 days, respectively. Experienced and novel groups at each age category were compared using ANOVA tests while considering the Blindness and Sex of the birds in the analysis using natural logarithm transformation on three dependent variables (Latency of the first head movement, first leg movement, and righting time). The back-transformation of marginal means and SE are reported in the Section 3. Mann–Whitney tests were also utilized to assess the effect of previous TI test experience on the variable of the ‘number of inductions required’.

## 3. Results

### 3.1. Body Weight

Heavier body weights within an age group have often been associated with a reduced number of induction attempts (a negative correlation), meaning heavier birds may enter tonic immobility more readily or quickly [34]. Body weight was recorded to determine whether blind and sighted birds differed in growth and to assess whether body size could potentially confound the interpretation of TI response. However, there was no significant correlation between body weight and any TI response variables. Blind chicks quickly learnt where the feed and water were using explorative behaviour (circular walking). Blindness did not have a significant effect on the body weight of the birds.

Results of a repeated-measures ANOVA test indicated that body weight of birds increased significantly at each age compared to the previous age, F(2,592) = 6822.71, *p* < 0.001 (37.33 ± 0.21, 320.01 ± 2.34, and 1015.67 ± 10.98 g at the ages of 1, 28, and 70 days, respectively). The main effect of Sex was also significant (F(1, 296) = 91.02, *p* < 0.001), as male chicks (497.33 ± 6.03 g) were heavier than female chicks (418.01 ± 5.72 g). Moreover, the interaction effect of Age and Sex was significant (F(2,592) = 76.88, *p* < 0.001; see Table 1).

### 3.2. Number of Inductions Required to Induce TI Response

The number of inductions required to induce a TI response was significantly different at different ages of the birds (χ^2^(3) = 23.39, *p* < 0.001). Mann–Whitney post hoc tests showed that more inductions were required to induce TI in birds at Day 2 (1.78 ± 0.16), Day 14 (1.42 ± 0.11), and Day 70 (1.44 ± 0.08) compared to Day 28 birds (1.08 ± 0.04).

The number of inductions required for inducing TI in blind birds was greater than that of sighted birds (U = 11,792.5, *p* = 0.005; 1.58 ± 0.09 vs. 1.31 ± 0.07, respectively). Males (1.47 ± 0.08) and females (1.41 ± 0.08) did not differ in the number of inductions required at different ages (U = 11,761.5, *p* = 0.12).

### 3.3. Latency of First Head Movement

Latency of first head movement and latency of righting time were both analyzed because they represent different stages of recovery from tonic immobility. Latency of first head movement reflects the initiation of recovery behaviour, whereas latency of righting time represents the termination of the tonic immobility state.

**Figure 3:**
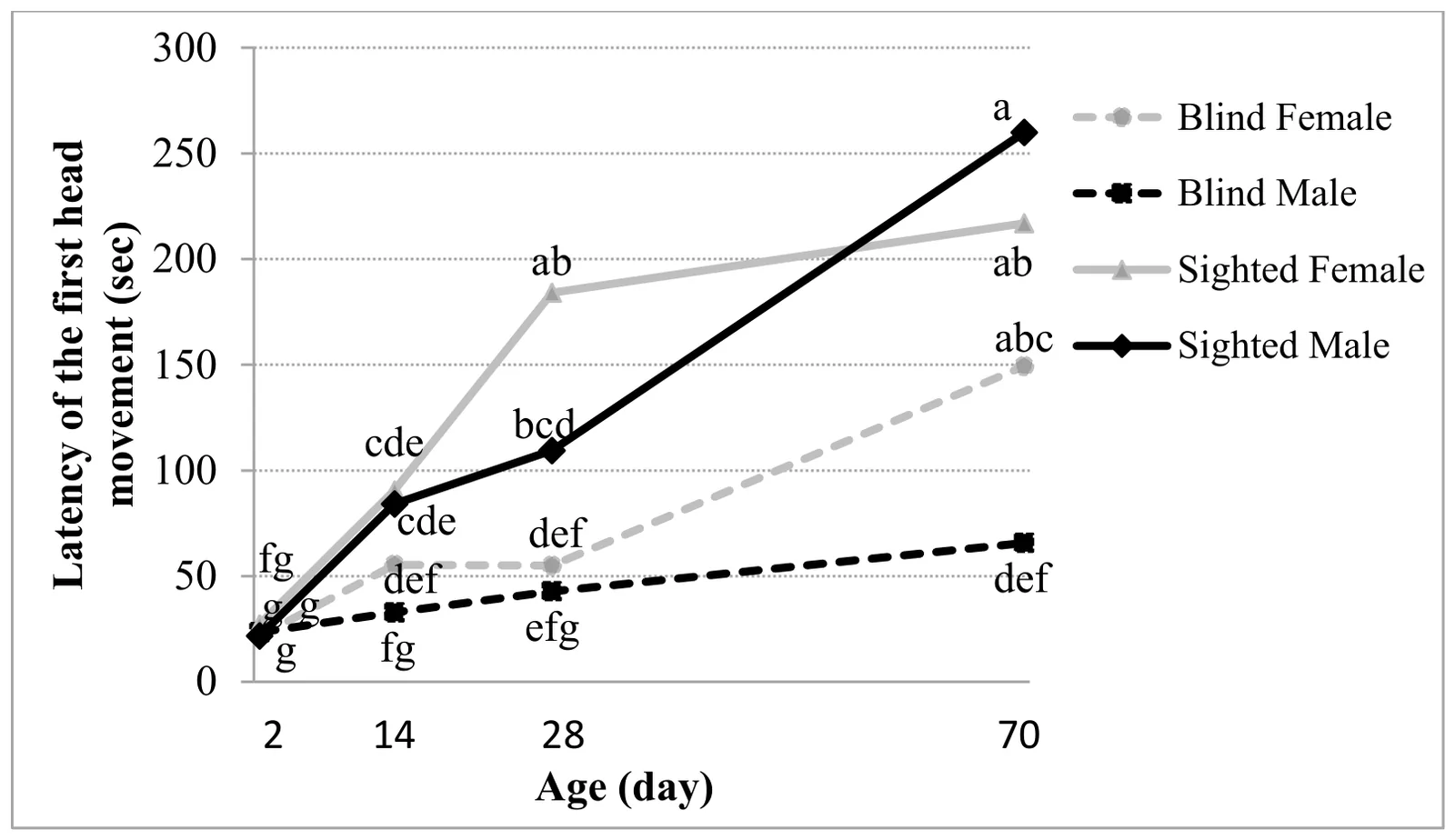
Latency of the first head movement (in sec) of chicks considering their Age, Sex and Blindness (^a–g^ dots with different letters are significantly (*p* < 0.05) different according to Sidak correction post hoc tests).

While the latency of first head movement increased as the birds aged, there was a significant (F(3, 303) = 2.65, *p* < 0.05; Figure 3) three-way interaction between Age, Blindness and Sex. At day 2, there was no significant difference among the four groups of birds. At Day 14, blind males and blind females did not differ in the latency of first head movement. Similarly, sighted males did not differ from sighted females. However, blind males had significantly shorter latency than both sighted males and females. At Day 28, the difference in latency of first head movement between blind and sighted birds became clearer. At Day 70, blind males had significantly shorter latency than the sighted birds. It is interesting to note that at this age, blind females had significantly longer latency than blind males, and this was not different from the sighted birds.

### 3.4. Latency of First Leg Movement

**Figure 4:**
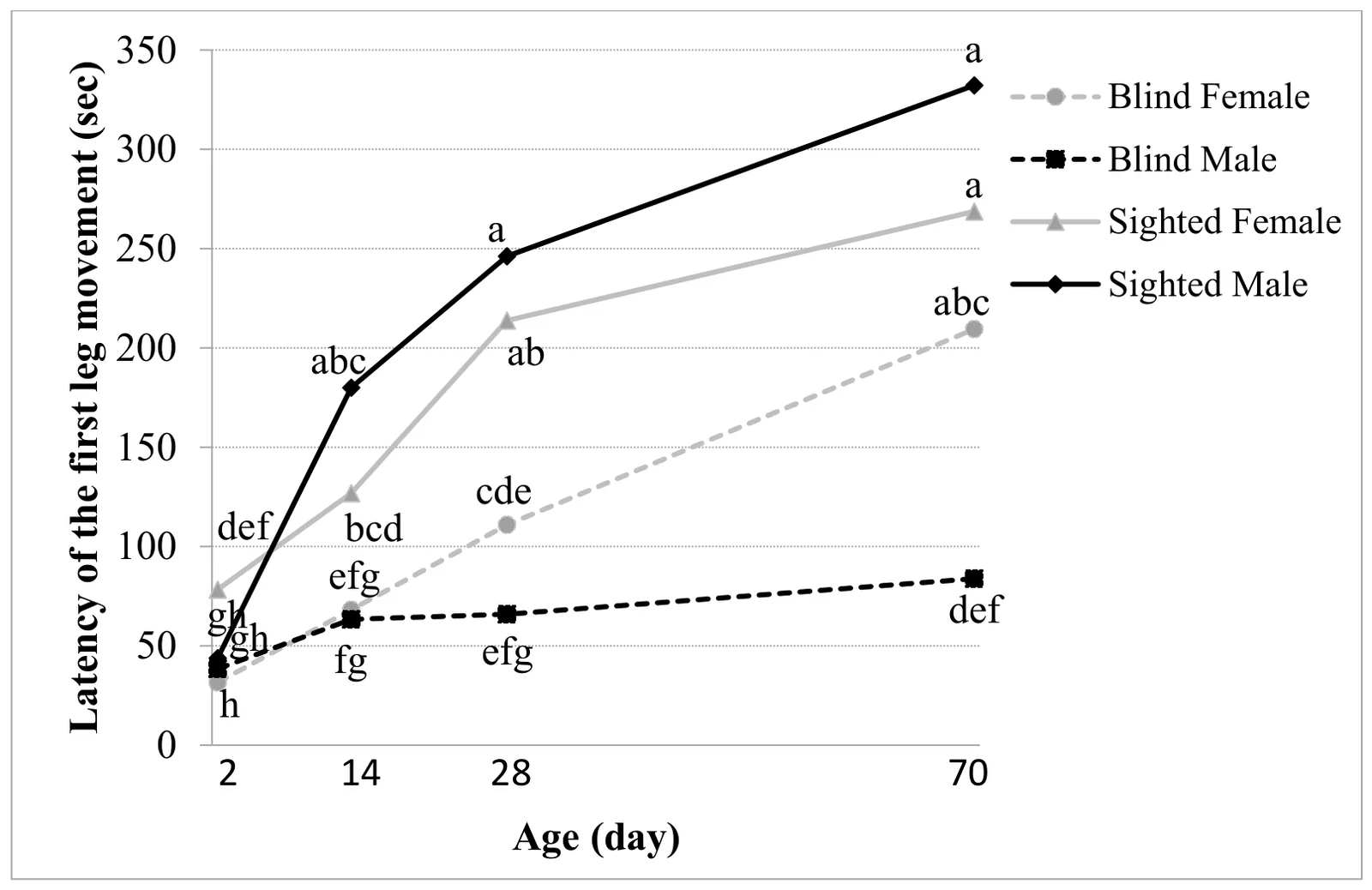
Latency of the first leg movement (in sec) of birds considering their Age, Sex and Blindness (^a–h^ dots with different letters are significantly (*p* < 0.05) different according to Sidak correction post hoc tests).

All birds had longer (F(3, 303) = 43.18, *p* < 0.001) latency of first leg movement as they aged. There was a significant (F(3, 303) = 4.47, *p* = 0.004; Figure 4) three-way interaction of Age, Blindness and Sex on the latency of first leg movement. At Day 2, sighted females had significantly longer latency of first leg movement than the other three groups, which were not significantly different among themselves. At Day 14, blind birds had significantly shorter latency than sighted birds. Blind females were not different from blind males and sighted females were not different from sighted males. At Day 28, the difference in latency between the blind and sighted birds became more pronounced. At Day 70, the blind males had significantly shorter latency than the sighted birds. Similarly to the latency of first head movement, blind females had significantly longer latency of first leg movement than blind males and were not different from the sighted birds.

### 3.5. Latency of Righting Time

**Figure 5:**
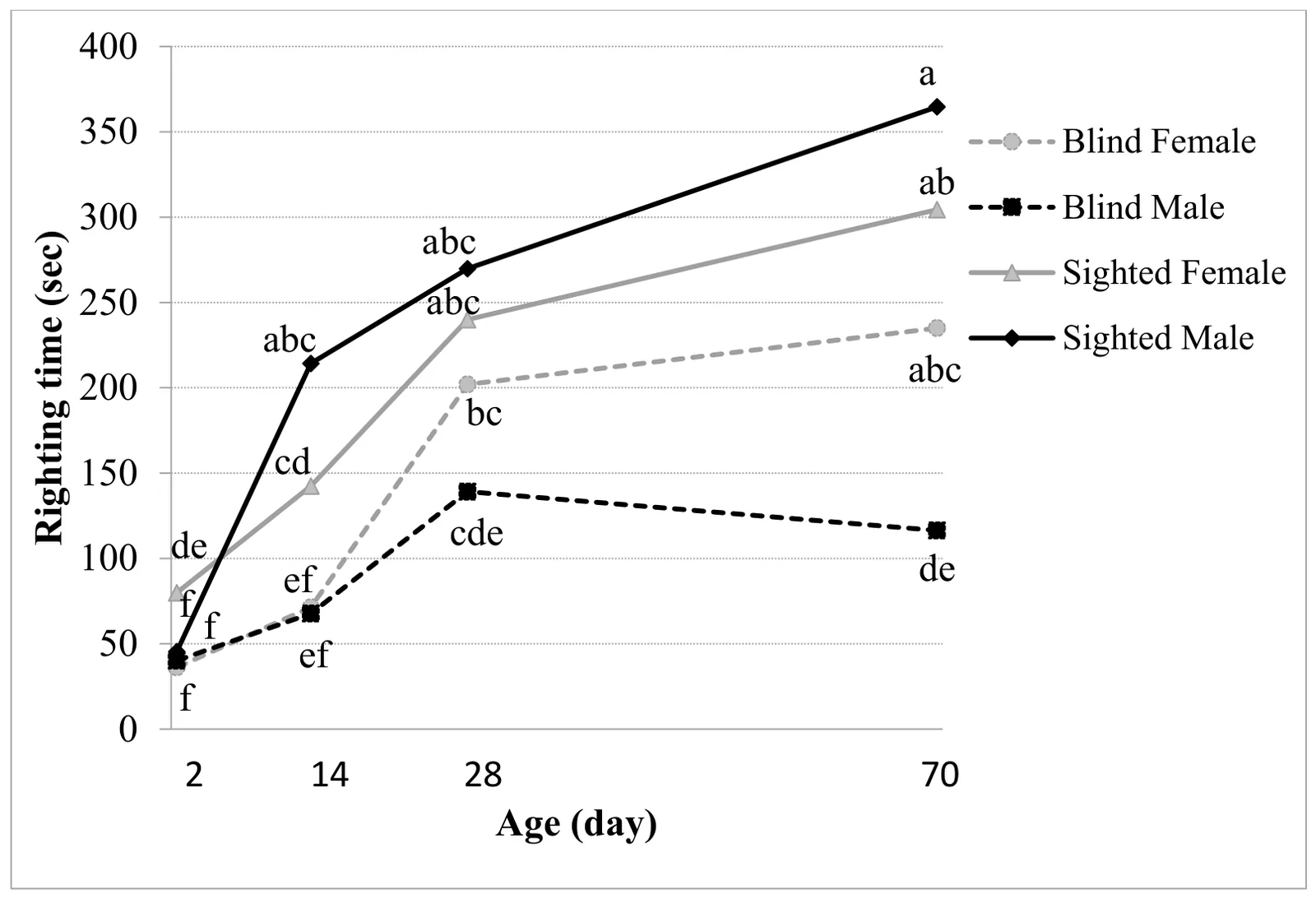
Latency of righting time (in sec) of birds considering their Age, Sex and Blindness (^a–f^ dots with different letters are significantly (*p* < 0.05) different according to Sidak correction post hoc tests).

All birds had significantly (F(3, 203) = 59.4, *p* < 0.001) longer latency of righting time as they aged. There was a significant (F(3, 303) = 3.23, *p* = 0.02; Figure 5) three-way interaction of Age, Blindness and Sex on the latency of righting time. At Day 2, sighted female chicks already took significantly longer to right themselves. At Day 14, sighted birds had significantly longer latency of righting time than blind birds. However, on Day 28, although the trend remained the same, there were no longer significant differences in latency of righting time among the four groups of birds. At Day 70, sighted males, sighted females, and blind females had significantly longer latency of righting time than blind males. There were no significant differences among sighted males, sighted females and blind females.

### 3.6. Effect of Prior TI Test Experience on Repeat TI Test

#### 3.6.1. Number of Inductions Required to Induce TI

At Day 28, experienced birds (1.22 ± 0.06) required significantly (U = 3315.50, *p* = 0.03; Mann–Whitney Test) more inductions than novel birds (1.08 ± 0.04). There was no significant difference between experienced and novel birds at Day 14 and Day 70.

#### 3.6.2. Latency of First Head Movement

**Figure 6:**
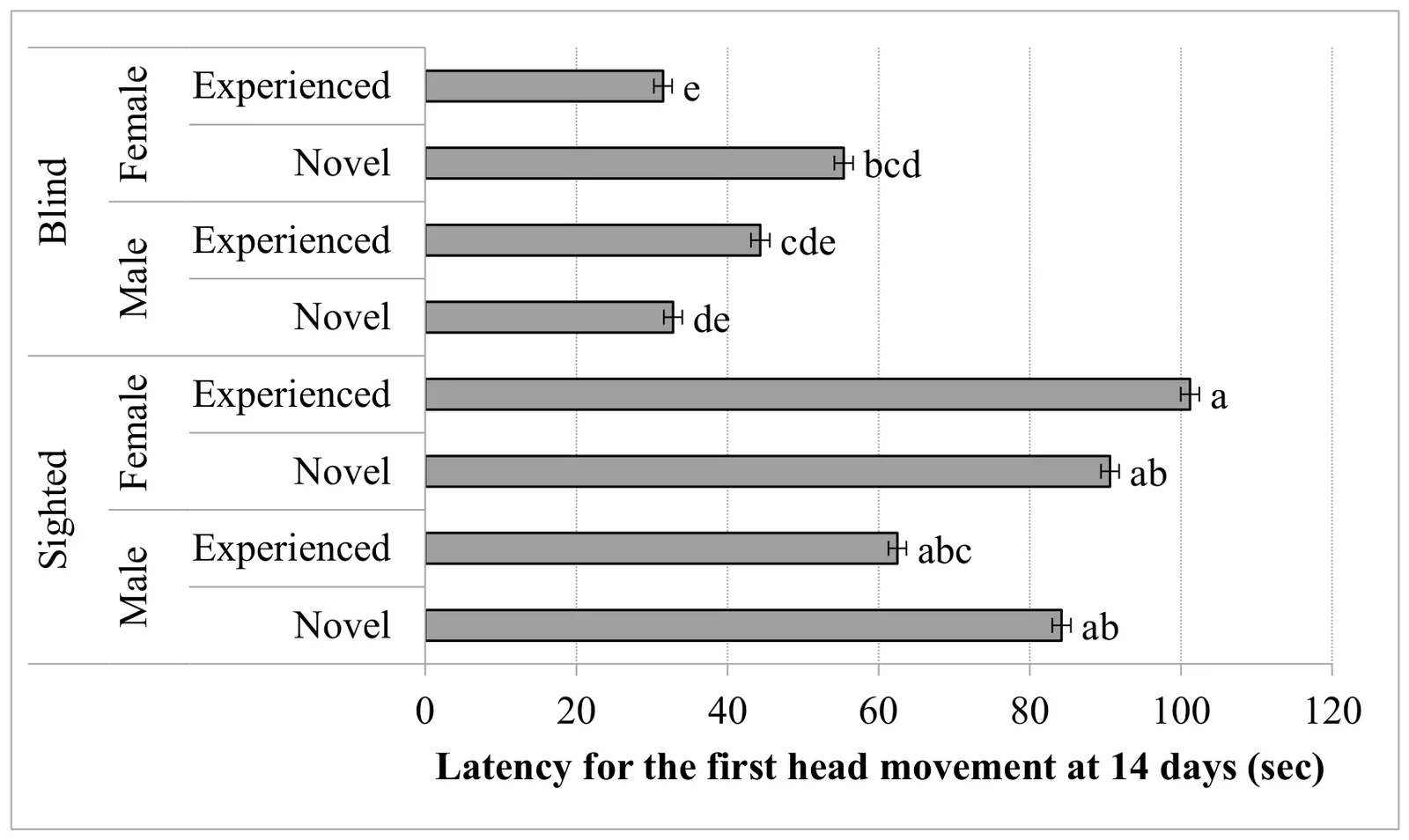
Three-way interaction of Blindness, Sex and Experience on the latency of the first head movement (in sec) at 14 days (bars with different letters are significantly (*p* < 0.05) different according to Sidak correction post hoc tests).

There was a significant (F(1, 150) = 5.22, *p* = 0.02) three-way interaction of prior Experience, Blindness, and Sex on latency for first head movement at Day 14 (Figure 6). There was no significant difference in latency between sighted males and females, whether they were experienced or novel. There was also no clear separation among the blind birds. On the whole, experienced birds (45.52 ± 1.11 s) had significantly (F(1, 150) = 28.46, *p* < 0.001) shorter latency than novel birds (102.57 ± 1.10 s).

#### 3.6.3. Latency of Righting Time

**Figure 7:**
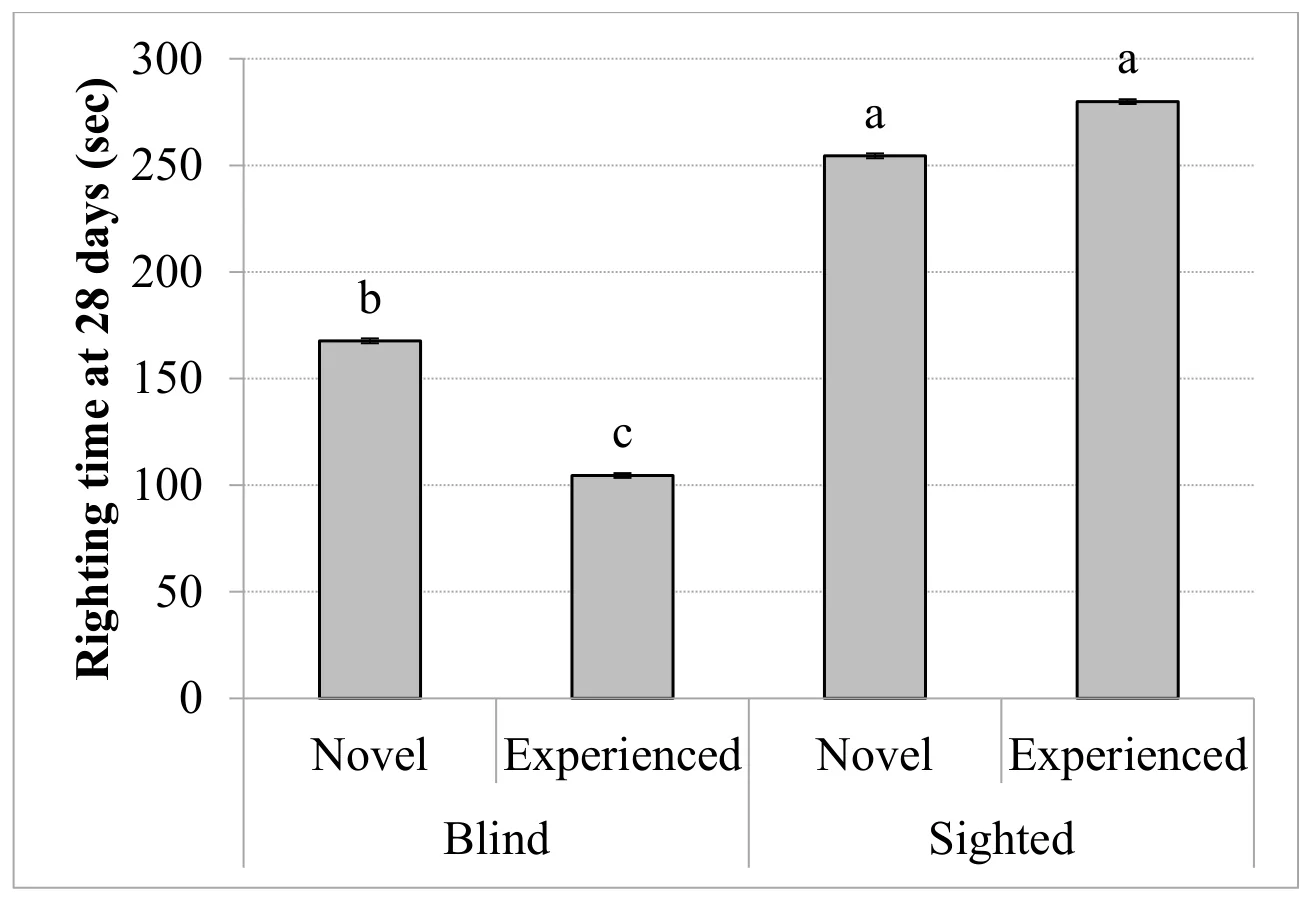
The righting time duration (in sec) considering Blindness and prior TI test experience at day 28 (^a–c^ bars with different letters are significantly (*p* < 0.05) different according to Sidak correction post hoc tests).

There was a significant (F(1, 160) = 5.56, *p* = 0.01) interaction between Blindness and prior Experience on the latency of righting time at Day 28. There was no significant difference between experienced and novel sighted birds. Experienced blind birds had shorter latency of righting time than novel blind birds (Figure 7). Overall, sighted birds had longer latency than blind birds.

### 3.7. Effect of the Time of Testing (Morning, Afternoon or Evening) on TI Test

The number of inductions required was significantly (*H*(2) = 7.50, *p* = 0.02; Kruskal–Wallis Test) higher in the morning tests (1.68 ± 0.12) compared to the afternoon (1.36 ± 0.08) and evening (1.39 ± 0.12) tests. Time of testing did not have a significant effect on Latency of first head movement, Latency of first leg movement, or Latency of righting time.

## 4. Discussions

Our study on the effects of genotype on TI in the development of fear response in chickens is unique. A recent literature search found no other studies of blindness and development of fear response in chickens. The *rc* chicken model provided a rare opportunity to compare the development of fear response in blind and sighted birds in the same flock. In addition, most studies on the development of fear response in chickens have been restricted to periods of less than 14 days to avoid the effect of multiple exposure. The housing of sufficient number of birds to avoid multiple exposure has been the obstacle [35].

The TI test is a well-validated fear test that can be used for domestic fowl [6,33,36]. Our results indicated that the TI duration of genetically blind Rhode Island Red chickens was significantly shorter than that of sighted birds of the same breed. Moreover, blind birds were less susceptible and needed more inductions to show TI response. This is an indication that blind birds may be less stressed and less frightened by handling compared to sighted birds [37].

Previous reports showed that vision-restricted hens had less aggressive and agonistic behaviour than normal-vision control laying hens [38]. When birds see a new object, they react, and novelty per se is a particularly potent fear stimulus or elicitor in birds [39]. Sighted chickens may have significantly more opportunities than blind birds for contact with novel stimuli during their lifetime and during TI tests. Therefore, it is not surprising that the blind chickens showed less of a fear response than their sighted counterparts.

It has also been suggested that chickens often perceive contact with humans as an alarming predatory encounter [40,41]. Two of the most common and potentially most frightening events encountered by domestic fowl are sudden changes in their environment and prompt exposure to unfamiliar human beings [39]. Visual contact with humans may be perceived as particularly threatening by birds [39]. Familiarity with a particular handler has shown to attenuate the TI response [12]. Researchers interested in the visual aspect of the fear response have restricted chicken vision by using poly-peepers or hoods [42]. Although they were successful in restricting straight-ahead vision to stop flock aggression, they could not totally block vision with the poly-peepers [38].

The reduced fear response in blind chickens is probably due to their inability to make visual contact with the researcher while the TI test was being performed. Blind chickens did not need to be caught. Mature blind hens often perform a “crouching” display (mating posture) when touched by human hands and can be picked up easily (Cheng, pers. obs). When carried in human hands, blind chickens remained calm and did not try to escape. Mature blind roosters were observed to start crowing while being carried. The possibility of sighted birds seeing and being caught by the experimenter may have contributed to the ease of induction and increased latencies of TI response in the present study.

### 4.1. Developent of Fear Response as Measured by TI

In our study, there was not a significant difference in latency for the first head movement of sighted and blind chicks on the first TI experiment day, when the birds were only two days old. Ratner and Thompson [18], and Salzen [19] reported that TI response is absent before 7 days of age. Salzen [19] explained that the TI response is physiologically immature at earlier ages. After the first week post-hatching, birds establish a familiar social environment and thus feel frightened by any threatening or novel factor that is not familiar to them.

Nakasai et al. [33] found that in native Japanese chickens, the TI duration in males was shorter than that in females at 2 days old, but longer than that in females at 15 days old. We found the same trend with our sighted birds. However, TI duration was not significantly different between our blind males and females at both ages.

In our study, older birds showed significantly longer TI responses compared to younger birds, and latency of the first leg movement and righting time reached their maximum levels at the third TI measurement day (Day 28). The onset of sexual maturity in chickens starts at around 4 weeks (28 days) of age and involves extensive physiological and metabolic changes [33,43]. For the sighted birds, we found no significant changes in the three measurements (Latency of first head movement, first leg movement, and righting time) of TI response over time (from Day 28 to Day 70) in either sex. For the blind birds, blind females had a longer TI response at Day 70 compared with Day 14. At Day 70, there were no significant difference between blind and sighted females in their TI response. Blind males showed no significant change in their TI response from Day 14 to Day 70. As a result, blind males had significantly shorter TI response than the other three groups at day 70.

Archer [43], studying six different genetic stocks of chickens, found that all mature males had a longer duration of TI response than all mature females. Campo et al. [44] further showed that mature females had shorter TI duration than mature males, but immature hens showed longer durations than immature males. Hens showing early sexual maturity had shorter TI reactions than hens with late sexual maturity. On the contrary, the TI duration was longer for the mature males compared with immature males [45].

The day a hen lays her first egg—known as the Age at First Egg (AFE)—is the definitive physiological marker for sexual maturity. Lighter, commercial breeds (like Leghorns) often mature faster, and start laying eggs at about 17–19 weeks of age. Heritage or heavy dual-purpose breeds may take slightly longer. Rhode Island Red hens, a dual-purpose breed, typically lay their first egg at between 18 and 22 weeks of age. Ali and Cheng [37] compared egg production of 20-week-old blind and sighted *rc* hens. AFE for blind hens was a few days earlier than AFE of sighted hens. In the following 2 months, blind hens produced 12.7% more eggs (hen-day production) while requiring 44.1 g less feed per bird per day compared to sighted hens.

Blindness in *rc* chickens is caused by a deletion in the *GC1* locus resulting in the disruption of cGMP (cyclic guanosine monophosphate) metabolism [32,46]. With a drop in cGMP, the photoreceptors cannot maintain their resting potential, recover from light exposure, process visual signals, and trigger photoreceptor apoptosis [47,48]. cGMP is also a crucial intracellular messenger in neuronal, muscle [49,50], and endocrine cells [51]. Sexual maturity and testosterone synthesis in male chickens are principally driven by the hypothalamic–pituitary–gonadal (HPG) axis via luteinizing hormone (LH) stimulation of the Leydig cells [52,53]. A deficiency of cGMP can affect testosterone production and reproductive functions, because cGMP signalling pathways play a regulatory role in the steroidogenic activity of Leydig cells and can delay male gonadal development and onset of sexual maturity [54].

Cerruti Sola et al. [55] reported that in 20-week-old blind *rc* male testes, the germinal layer showed delayed maturation and poor differentiation of germinal cells. There was a complete lack of spermatocytes, spermatids, and spermatozoa. Arshami and Cheng [56] compared testicular development and sperm production between 22-week-old blind and sighted *rc* males. Blind males had significantly lower volume of ejaculate, total sperm count per ejaculate, % sperm motility, % live sperm, and sperm metabolic activity after ejaculation than sighted males. Histological examination of the testes found that blind males had significantly reduced seminiferous tubule length and diameter, with significantly lower % of seminiferous tubules filled with elongated sperm compared with sighted male testes. These results indicated delayed sexual maturation in the blind males. Campo et al. [44] found that the TI duration was longer for mature males compared with immature males.

In female avian tissues, the ductus arteriosus and hypothalamus, estrogen (such as 17β-estradiol) interacts with membrane-associated receptors to stimulate nitric oxide (NO) production, which in turn activates the enzyme soluble guanylyl cyclase (sGC) to rapidly synthesize intracellular cGMP [57]. This may compensate for the cGMP deficiency caused by the *rc* mutation. Thus, it seems that blind *rc* hens may bypass the cGMP deficiency that would affect their rate of sexual maturity and egg production.

Despite the lack of rod and cone photoreceptors, blind *rc* chickens can still perceive light via the inner retina [46,58]. They still maintain a circadian system of pineal melatonin secretion [59]. Behavioural studies have shown that blind chickens demonstrate entrainment of feeding rhythms to the light–dark cycle [60]. Our study found that both the blind and sighted chickens required significantly more inductions to induce TI in the morning compared with both the afternoon and evening.

In this study, novel birds showed longer latency for the first leg movement at the age of 14 days compared to the birds with previous TI measurement experience, and experienced birds required more inductions at Day 28 (were less susceptible) compared to novel birds. Blind experienced birds also had a shorter righting time compared to other birds in our study at the age of 28 days. Similarly, Ratner and Thompson [18] reported that previous tests and related handling experience reduced TI response duration and increased the number of required inductions, and Zulkifini et al. [18] indicated that visual contact with the experimenter during the first 3 weeks after hatching decreased the duration of TI response. Gilman [12] also reported that previous handling experience before TI testing decreased the susceptibility of birds to TI, and the results of 40 repeated TI tests over 21 days indicated that experience decreased the susceptibility and the duration of TI response.

### 4.2. Pleiotropic Effect of the Mutation

In brain cells, cGMP acts as a critical second messenger bridging extracellular signals to neuronal plasma membrane functions and gene expression. It regulates synaptic plasticity, memory formation, and ion channel activity [61]. When a chicken enters TI, central cholinergic pathways (using acetylcholine) are highly active, maintaining motor inhibition and the fear state [62]. cGMP acts as a critical intracellular secondary messenger in these cholinergic pathways. With a deficiency of cGMP due to the mutation, the fear signal cannot be translated into the full physical manifestation of the immobility response [63]. It is therefore likely that in addition to blindness minimizing the perception of fear and the development of a fear response, the mutation in *rc* chickens also acted directly on the brain to minimize the TI response [59,64].

## 5. Conclusions

The objective of this study was to examine the effect of blindness on the development of a fear response in rc chickens using the TI test. We rejected both null hypotheses but concluded that differences in TI between blind and sighted *rc* chickens may not necessarily be attributed solely to the absence of visual input.

Age-related comparisons in this study were conducted using separate cohorts of birds tested for the first time at each age to avoid the potential confounding influence of previous TI experience on behavioural responses. However, as with any cohort-based design, a contribution of cohort-specific variation cannot be completely excluded.

Our study provides valuable data and references for further understanding the developmental characteristics of fear-related behaviours and welfare in domestic chickens. The *rc* chicken is also a model of Leber’s congenital amaurosis (LCA), a congenital blindness, in humans because both carry mutations in the same gene [32,46]. Our study demonstrated direct association of physiological abnormalities with this mutation and provides insight into systemic abnormalities that are often associated with LCA. Intervention targeting these abnormalities will enhance the efficiency of gene therapy (https://www.pennmedicine.org/news/improvement-in-sight-seen-after-gene-therapy-trial; accessed on 28 August 2026) in improving the quality of life of LCA sufferers.

## Figures and Tables

**Table 1:** Body weights of male and female birds at different ages (in g).

Age of Birds	Male (g)	Female (g)
Day 2	37.60 ± 0.31 ^e^	37.07 ± 0.29 ^e^
Day 28	338.24 ± 3.40 ^c^	301.79 ± 3.23 ^d^
Day 70	1116.17 ± 15.93 ^a^	915.17 ± 15.13 ^b^

^a–e^ Marginal means sharing the same superscript letter are not significantly different, whereas marginal means with different superscript letters differ significantly (Sidak-adjusted *p* < 0.05).

## Data Availability

The raw data supporting the conclusions of this article will be made available by the authors on request.

## Abbreviations

TI	Tonic Immobility
cGMP	Cyclic guanosine monophosphate
GC1	Photoreceptor guanylate cyclase
ANOVA	Analysis of variance
AFE	Age at first egg
